# Biocrusts Modulate Climate Change Effects on Soil Organic Carbon Pools: Insights From a 9-Year Experiment

**DOI:** 10.1007/s10021-022-00779-0

**Published:** 2022-09-27

**Authors:** Paloma Díaz-Martínez, Marco Panettieri, Pablo García-Palacios, Eduardo Moreno, César Plaza, Fernando T. Maestre

**Affiliations:** 1 Departamento de Biología y Geología, Física y Química Inorgánica, Universidad Rey Juan Carlos, 28933 Madrid, Spain; 2 Instituto de Ciencias Agrarias (ICA), CSIC, Serrano 115 bis, 28006 Madrid, Spain; 3 Departamento de Química Agrícola y Bromatología, Facultad de Ciencias, Universidad Autónoma de Madrid, 28049 Madrid, Spain; 4 Instituto Multidisciplinar Para el Estudio del Medio “Ramón Margalef”, Universidad de Alicante, Alicante, Spain; 5 Departamento de Ecología, Universidad de Alicante, Alicante, Spain

**Keywords:** carbon cycling, soil organic matter, particulate-associated organic carbon, mineral-associated organic carbon, nuclear magnetic resonance, biocrusts, climate change

## Abstract

Accumulating evidence suggests that warming associated with climate change is decreasing the total amount of soil organic carbon (SOC) in drylands, although scientific research has not given enough emphasis to particulate (POC) and mineral-associated organic carbon (MAOC) pools. Biocrusts are a major biotic feature of drylands and have large impacts on the C cycle, yet it is largely unknown whether they modulate the responses of POC and MAOC to climate change. Here, we assessed the effects of simulated climate change (control, reduced rainfall (RE), warming (WA), and RE + WA) and initial biocrust cover (low (< 20%) versus high (> 50%)) on the mineral protection of soil C and soil organic matter quality in a dryland ecosystem in central Spain for 9 years. At low initial biocrust cover levels, both WA and RE + WA increased SOC, especially POC but also MAOC, and promoted a higher contribution of carbohydrates, relative to aromatic compounds, to the POC fraction. These results suggest that the accumulation of soil C under warming treatments may be transitory in soils with low initial biocrust cover. In soils with high initial biocrust cover, climate change treatments did not affect SOC, neither POC nor MAOC fraction. Overall, our results indicate that biocrust communities modulate the negative effect of climate change on SOC, because no losses of soil C were observed with the climate manipulations under biocrusts. Future work should focus on determining the long-term persistence of the observed buffering effect by biocrust-forming lichens, as they are known to be negatively affected by warming.

**Supplementary Information:**

The online version contains supplementary material available at 10.1007/s10021-022-00779-0.

## Introduction

Drylands include arid, semiarid, and dry-subhumid ecosystems and altogether represent about 41% of the global land surface area (Cherlet and others 2018). Despite their reduced soil organic carbon (SOC) content compared with more mesic environments, because of their vast extension they represent 32% of the global SOC pool, which is equivalent to 646 Pg of organic C (Plaza and others 2018). Drylands also play a critical role in regulating the variability of the global terrestrial C sink (Ahlström and others 2015) and thus are of paramount importance when forecasting the climate of the future and its impact on terrestrial ecosystems (Safriel and others 2005). The rising temperatures and the concomitant increasing aridity projected by climate models in drylands over this century (Feng and Fu 2013) may reduce plant C inputs into the soil and increase soil organic matter decomposition (Davidson and Janssens 2006; Maestre and others 2015). This not only may increase the flux of CO_2_ to the atmosphere and reinforce climate change, but also may accelerate land degradation and desertification (Berdugo and others 2020; Huang and others 2016).

The top surface of dryland soils, where SOC largely accumulates (FAO 2015; Thomas 2012), is often dominated by biocrusts (soil communities mainly composed of cyanobacteria, algae, bryophytes, lichens, and fungi in varying proportions that create a surface crust of soil particles of few millimeters bound together by organic materials; Belnap and others 2016). Biocrusts have been estimated to cover about 12% of the Earth's terrestrial surface (Rodriguez-Caballero and others 2018) and can be particularly abundant in arid and semiarid regions (Maestre and others 2021). In addition to playing essential roles in multiple soil functions such as water and nutrient cycling, biological weathering and soil stabilization (Belnap and others 2016; Concostrina-Zubiri and others 2021; Delgado-Baquerizo and others 2016), these communities fix over 2.6 Pg year^−1^ of atmospheric C globally (Elbert and others 2012) and play a key role in soil organic C cycle across global drylands. For example, extracellular polysaccharides from cyanobacteria promote nutrient absorption by soil particles (Mager and Thomas 2011) and increase soil stability, reducing soil erosion (Bowker and others 2018). Growing evidence shows that climate change will affect biocrust communities worldwide (Belnap and others 2016; Baldauf and others 2021; Ladrón de Guevara and others 2018; Reed and others 2019). Increases in aridity linked to climate change are expected to considerably decrease the cover and shift the distribution of biocrusts in drylands (Rodriguez-Caballero and others 2018), hampering the ecosystem services provided by these communities.

The impact of climate change on SOC content in soils covered by biocrusts will also be determined by their influence on SOC stability. Traditionally, SOC stability was attributed to its molecular composition, but now we know that it also depends on its interactions with soil minerals, which play a major role by posing physical and chemical barriers to microbial decomposition (Kleber and others 2021). For this reason, the separation of SOC into particulate organic C (POC) and mineral-associated organic C (MAOC) is particularly useful to understand SOC dynamics and responses to climate change (Lavallee and others 2020). The POC fraction is mainly in the form of undecomposed or partially decomposed plant and fungi structural organic compounds with low N content (Golchin and others 1994; Cotrufo and Lavalle 2022). In contrast, the MAOC fraction consists of relatively lower molecular weight compounds mainly produced by microorganisms (for example, cellular and extracellular microbial compounds; Bradford and others 2013; Kleber and others 2015; Liang and others 2019;). In addition, being chemically bound to soil minerals, MAOC is more protected from decomposition than POC (Lavallee and others 2020). As a result, POC has been shown to have a faster turnover cycle, higher vulnerability to alterations, and higher responsiveness to management than MAOC (Poeplau and others 2018; Wander 2004; Cotrufo and Lavallee 2022). Also, because of their different compositions, arrangement in the soil mineral matrix, and cycling rates, POC and MAOC exert different functions. Current paradigms indicate that POC is more closely associated with soil biological activity and nutrient provision to plants, whereas MAOC provides a more stable C pool for climate regulation, plant nutrient retention, and water holding capacity (Wander 2004; Wood and others 2016). Climate change has been suggested to alter the various mechanisms of SOC protection from microbial decomposition (Conant and others 2011; Davidson and Janssens 2006; Groffman and others 2001). However, there are few studies evaluating the effect of climate change on the stability of C in drylands using C fractionation (Bai and others 2020; He and others 2012; Link and others 2003; Song and others 2012), and its molecular composition has been barely assessed so far (Puissant and others 2017; Schnecker and others 2016). To the best of our knowledge, no previous study has evaluated the joint effects of biocrusts and climate change on SOC fractions. Doing so could shed novel insights on the effects of climate change on SOC stability and functioning in biocrust-dominated drylands and would advance our capacity to quantify the feedback between terrestrial C cycle and climate change.

Using a climate change experiment located in a semiarid ecosystem in the center of Spain, Maestre and others (2013) observed a decrease in biocrust cover paralleled by an increase in SOC content in high initial biocrust cover soils after four years of warming. We examined whether the increases in SOC content observed in this experiment lasted for 9 years and evaluated the impacts of biocrust development and simulated climate change on C stability and its vulnerability to simulated climate change. To do so, we assessed whether biocrusts regulate the effects of climate change (ambient conditions, 35% rainfall reduction, 2–3 ºC warming, and the combination of both) on SOC, POC and MAOC contents, and molecular composition of POC using solid-state ^13^C nuclear magnetic resonance.

## Materials and Methods

### Site and Experimental Design

The field experiment used in this work is located at the Aranjuez Experimental Station, in central Spain (40° 02′ N, 3° 32′ W, 590 m above sea level). The climate is semiarid Mediterranean, with warm dry summers and moist cool winters. The mean annual temperature is 15 ºC, and the mean annual precipitation is 349 mm. Perennial vegetation cover is below 40% and mainly consists of grasses, such as *Stipa tenacissima*, and small shrubs, such as *Helianthemum squamatum* and *Gypsophila struthium*. The bare areas between perennial vegetation are colonized by a well-developed biocrust community that covers about 32% of the soil surface and is dominated by several lichen species, such as *Diploschistes diacapsis*, *Squamarina lentigera*, *Fulgensia subbracteata,* and *Psora decipiens*, and mosses such as *Pleurochaete squarrosa* and *Didymodon acutus* (Castillo-Monroy and others 2011; Maestre and others 2013). The soil is a Gypsiric Leptosol (IUSS Working Group WRB 2015), with pH values around 7 (Maestre and others 2013).

The experiment was set up in 2008 and has a full factorial design, with three factors, each with two levels, and ten replicates per treatment applied in 1.25 × 1.25-m plots distributed in the interspaces of plants randomly and separated at least 1 m from each other. The factors are biocrust cover at the beginning of the experiment (< 20% vs. > 50%, or low vs. high), rainfall exclusion (RE, control vs. rainfall exclusion), warming (WA, control vs. warming), and the combination of both (RE + WA). The RE scenario was simulated with passive rainfall shelters based on the design of Yahdjian and Sala (2002). Every rainfall shelter is composed of three methacrylate grooves (see extended data Figure 1) connected to plastic bottles that accumulate the excluded water. Most climate models foresee 10–50% reductions in the total amount of rainfall during spring and fall in the next decades in our study area (Escolar and others 2012). To simulate these conditions, each methacrylate groove has an area of 1.44 m^2^ (1.2 × 1.2 m) and a mean height of 1 m, covers approximately 37% of the surface, and reduces the total amount of rainfall reaching the soil surface by 33% on average. Open-top chambers (OTCs) were used to simulate a temperature scenario similar to that forecast by general circulation models, that is, a temperature increase of 2–3 °C for 2040–2070 (De Castro and others 2005). The OTCs consist of methacrylate sheets (40 × 50 × 32 cm) forming a hexagon and suspended 3–5 cm over the ground by a metal frame to avoid overheating by allowing air circulation. Methacrylate ensures 92% transmittance in the visible spectrum and very low emission in the infrared wavelength according to the manufacturer (Decorplax S.L.). See Escolar and others (2012) and Maestre and others (2013) for additional details on the experimental design and microclimatic impacts of OTCs and rainfall shelters.

**Figure 1:**
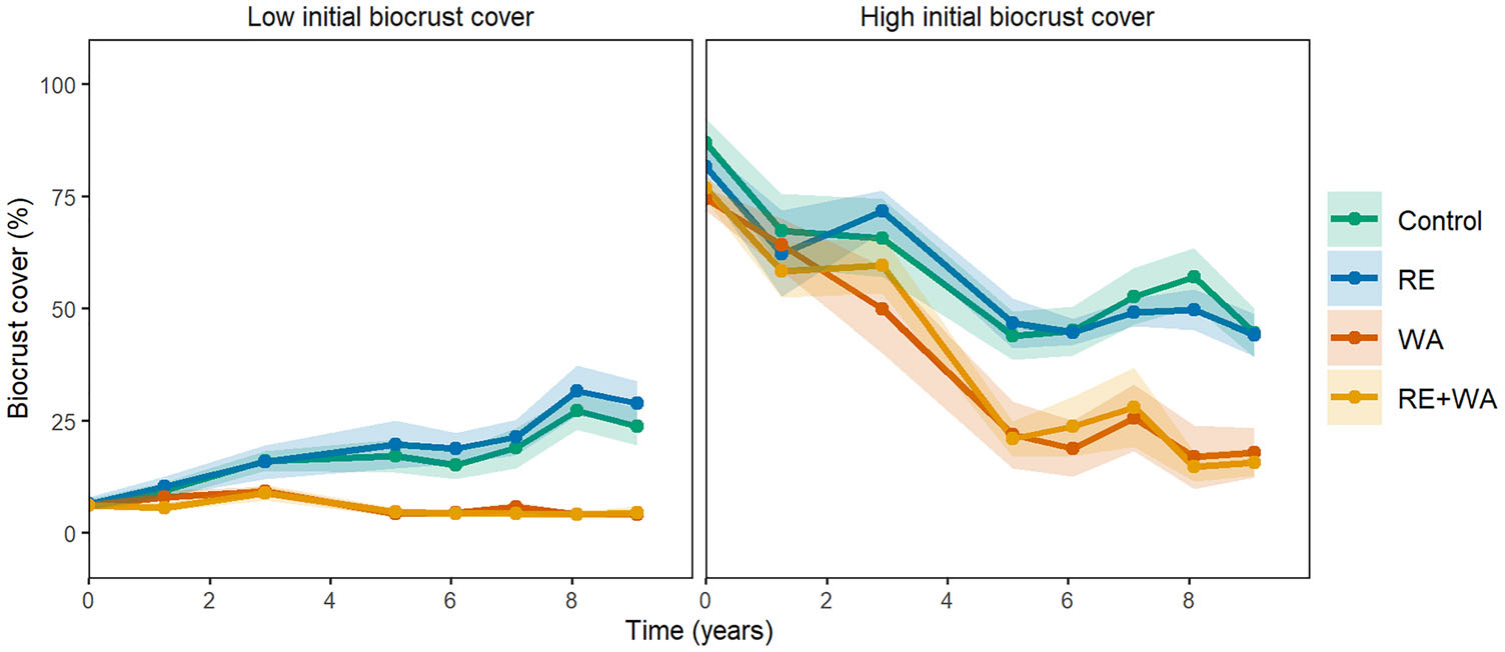
Evolution of biocrust cover (mean ± standard error, *n* = 10) in soils with low and high initial biocrust cover, as affected by ambient conditions (control), rainfall exclusion (RE), warming (WA), and the combination of both (RE + WA).

### Biocrust Cover Measurements

Biocrust cover was monitored within 20-cm-diameter PVC rings permanently installed one in each plot at the beginning of the experiment and after 1, 3, 5, 6, 7, 8, and 9 years. We used photographs and the software GIMP (http://www.gimp.org to map biocrust areas), and ImageJ (http://rsb.info.nih.gov/ij/, to calculate the size of biocrust-covered areas) (Maestre and others 2013). Biocrust cover measurements thus correspond to the area covered by the visible components of the biocrust community, namely lichens and bryophytes, of the 10 plots. This is a common and non-destructive method for measuring biocrust cover (Chamizo and others 2021; Wang and others 2021), but only considers visible biocrust components.

### Soil Sampling and Organic C Fractionation

Soil samples were collected from the top 0–1 cm from five randomly selected plots per treatment outside the PVC rings at the beginning of the experiment and 9 years later (June 2017). In the laboratory, the soil samples were passed through a 2-mm sieve and air-dried after carefully removing visible biocrust components.

SOC was fractionated into free light, occluded light, and mineral-associated organic C (MAOC) pools by the densimetric scheme of Golchin (1994). Briefly, 10 g of soil and 50 mL of NaI (at a density of 1.85 g mL^−1^) were shaken rotationally for 30 s with at 1 revolution s^−1^ within a centrifuge tube. The mixture was then centrifuged, and the floating free light C fraction separated from the heavy fraction (MAOC) by suction and filtration. The occluded light C was separated from the MAOC by a second-density separation after ultrasonic disruption of the aggregates in the heavy fraction with an energy input of 1500 J g^−1^. The recovered fractions were oven-dried at 60 ºC, weighed, and ground with a ball mill.

Total organic C content in soil samples and organic C fractions was determined by dry combustion with a Thermo Flash 2000 NC Soil Analyzer. Carbonates were removed before the analysis by acid fumigation (Harris and others 2001). The content of POC in the soil was calculated as the sum of the contents of occluded light C (< 5% of total SOC content) and free light C (Golchin and others 1994; Lavallee and others 2020).

We mixed the corresponding replicates of free light C and analyzed them by solid-state ^13^C cross-polarization at the magic angle spinning (CP-MAS) nuclear magnetic resonance (NMR). We focused this molecular compositional analysis on the free light organic C (POC) because this fraction has the fastest turnover (from less than 10 years to decades) and thus is arguably the most responsive to climate alterations (Lavallee and others 2020). The use of composite samples in NMR analyses in soil science is a common practice to reduce analytical times and costs, which takes advantage of the technical stability and reproducibility of the results provided by NMR spectroscopy. The NMR spectra were acquired on a Bruker AV 400 MHz wide-bore spectrometer equipped with a 4-mm ^1^H/X/Y MAS probe, operating at a MAS frequency of 10,000 Hz, a ramp-CP contact time of 3 ms, a recycle delay of 4 s, and approximately 10,000 scans were acquired for each spectra. The free induction decay signal was digitized and multiplied by an exponential function corresponding to 60 Hz line broadening in the final transformed spectrum. The spectra were then baseline corrected, calibrated relative to adamantane, and integrated into the following chemical shift regions: alkyl-C (0–45 ppm), *N*-alkyl/methoxyl C (45–60 ppm), *O*-alkyl C (60–90 ppm), anomeric C (90–110 ppm), aryl C (110–145 ppm), heteroaromatic C (145–165 ppm), carboxyl/amide C (170–225 ppm Knicker 2011; Lüdemann and Nimz 1973). Signals at 168 ppm due to carbonates were excluded from data treatment. The NMR spectra were treated and integrated using MestReNova software (version 12.0.4–22,023 ©Mestrelab Research S.L. 2018). To assess significant differences in composite samples analyzed with ^13^C NMR, Baldock and Smernik (2002) proposed a 2% difference in the area of spectral regions as limit of significance for spectra with high signal-to-noise ratios, while Diekow and others (2005) set up specific limits for each spectral region ranging from 2.5 to 8.3%.

### Data Analysis

Analyses were conducted separately for plots with low and high biocrust cover since data visual inspection and preliminary analyses showed that biocrust cover had important interactive effects with warming and/or rainfall exclusion (RE) on many of the response variables measured (Supplementary Table 1). This was also consistent with observations from Maestre and others (2013). The main and interaction effects of climate and time on biocrust cover were tested by linear mixed-effects models. Both a slope (time) and intercept (PVC ring) were incorporated in the random term of the model to account for the temporal autocorrelation because of the repeated measures on the same 10 plots over time (Bates and others 2015), and autocorrelation plots were used to check the independence of the residuals. The analyses of variance (ANOVAs) for the fitted linear mixed-effects models were conducted using the Satterthwaite's method for denominator degrees-of-freedom and F-statistic estimation. We used data from the 10 plots and transformed biocrust cover data using natural-logarithm to reduce their skewness.

To estimate how simulated climate change affected soil C variables throughout the duration of the experiment, we calculated the absolute effect size (A_e_) as C_9_–C_0_ where C_0_ and C_9_ are the values at the beginning of the experiment and nine years later, respectively. Because we selected random plots at the beginning, and at the end of the period, the evaluated C values do not correspond to the same plots, so A_e_ was calculated as C_9_ minus the mean C_0_ for each treatment (*n* = 5). The effects of climate change on the contents of SOC, POC, and MAOC (*n* = 40) at the beginning of the experiment, after 9 years, and on A_e_ were analyzed by linear models, or by Kruskal–Wallis and Dunn’s tests when the assumptions of normality and homoscedasticity of the residuals were not met. Normality and homoscedasticity were examined using Shapiro–Wilk and Levene’s and tests, respectively. All the data visualization and analyses were performed using R version 4.1.1 (R Core Team 2021) and the R packages tidyverse _1.3.1(Wickham 2017), readxl _1.3.1(Wickham and others 2019), lme4_ 1.1–27 (Bates and others 2015), lmerTest _3.1–3 (Kuznetsova and others 2015), dunn.test_1.3.5 (Dinno and Dinno 2017), and patchwork _1.1.1 (Pedersen 2020).

## Results

### Biocrust Cover

We found a significant interaction between the effects of climate change and time on biocrust cover, both in plots with low (F(3) = 12.9, *P* < 0.001) and high (F(3) = 4.32, *P* = 0.011) initial biocrust cover. In plots with low initial biocrust cover, biocrust cover in the control and RE treatments increased from 6% at the beginning of the experiment to 24 and 29%, respectively, after 9 years, whereas biocrust cover remained constant in WA and RE + WA during the entire experimental period (Figure 1). In plots with high initial biocrust cover, biocrust cover declined over time, especially in WA (from 74 to 18%) and RE + WA (from 77 to 16%) treatments, but also in RE (from 82 to 44%) and control (from 86 to 44%) conditions (Figure 1).

### Total, particulate, Mineral-Associated Organic C Contents

**Figure 2:**
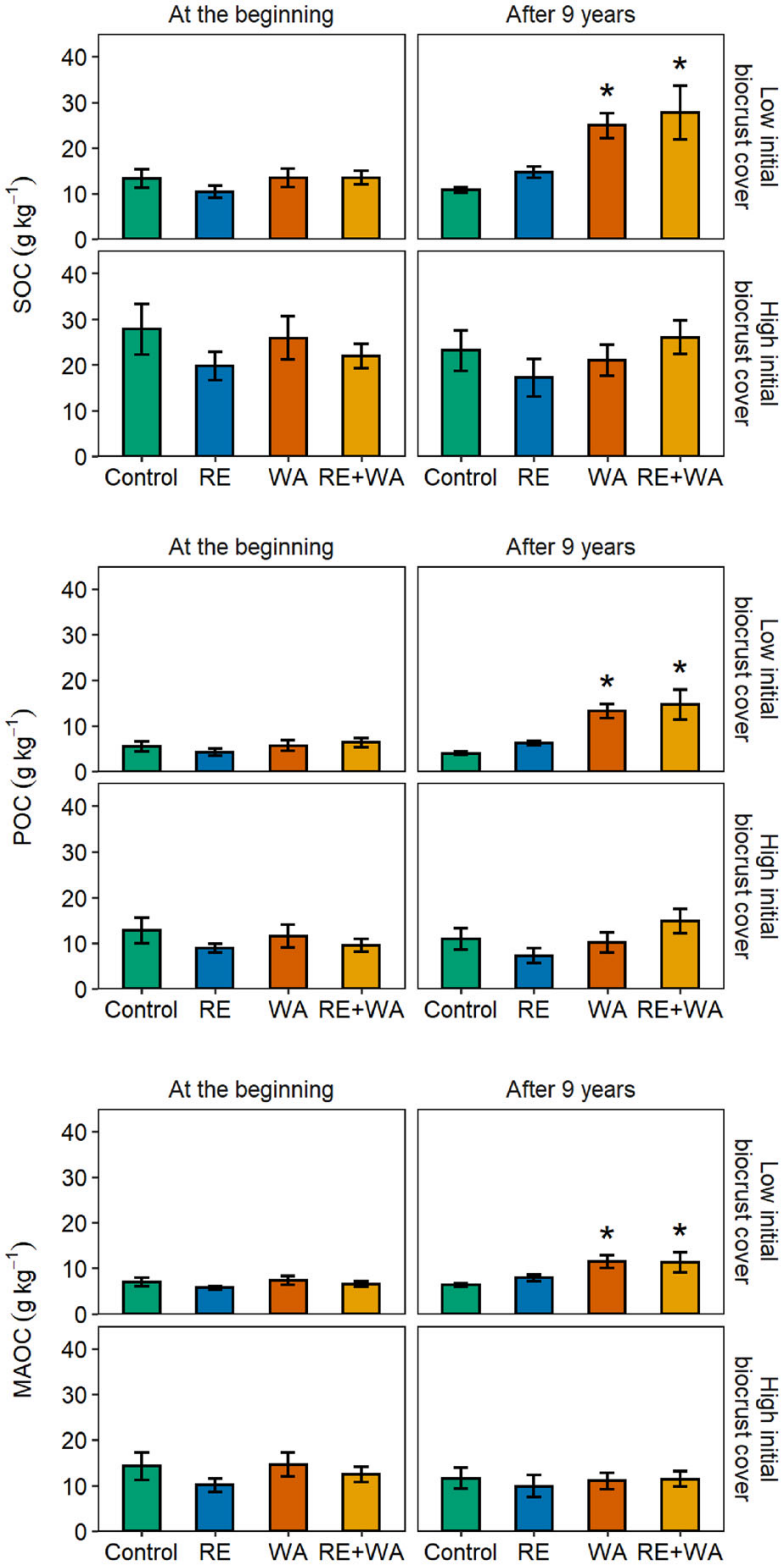
SOC, particulate organic C (POC), and mineral-associated organic C (MAOC) contents (mean ± standard error, *n* = 5) in soils with low and high initial biocrust cover, at the beginning of the experiment and after 9 years of ambient conditions (control), rainfall exclusion (RE), warming (WA), and the combination of both (RE + WA). *, significantly different from control (*P* < 0.05).

**Figure 3:**
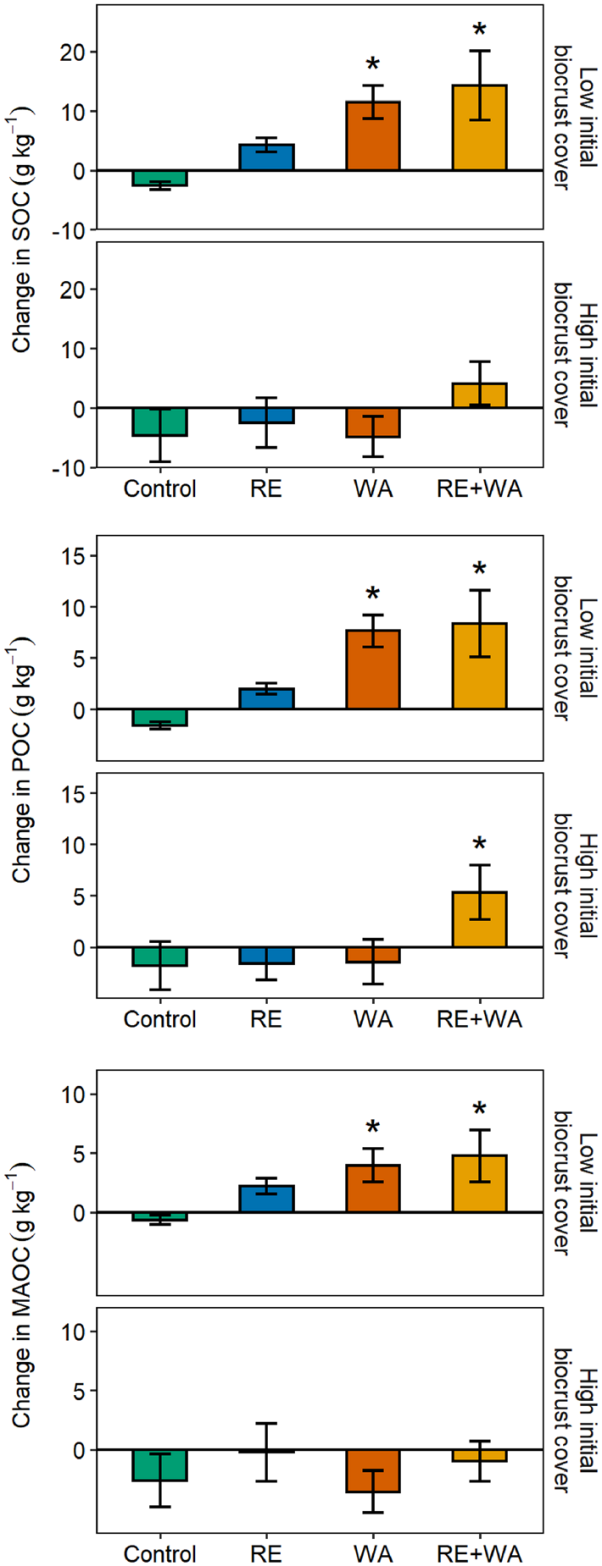
Changes in SOC, particulate organic C (POC), and mineral-associated organic C (MAOC) contents (mean ± standard error, *n* = 5) in soils with low and high initial biocrust cover after 9 years of ambient conditions (control), rainfall exclusion (RE), warming (WA), and the combination of both (RE + WA). *, significantly different from control (*P* < 0.05).

At the beginning of the experiment, the contents of SOC, POC and MAOC were higher in plots with high initial biocrust cover compared to those with low cover (Figure 2), and the percentage of the POC fraction in the total SOC pool was slightly lower (43%) than those of the MAOC fraction (55%) (Table 1). After nine years, soils with low initial biocrust cover subjected to WA and, especially to RE + WA, had significantly greater SOC, POC and MAOC contents than those under RE and ambient conditions, whereas soils with high initial biocrust cover were not affected by any of our climate change treatments (Figure 2). In plots with low initial biocrust cover and with respect to initial levels, SOC contents increased by 186% after WA and by 206% after RE + WA, POC increased by 235% after WA and by 232% after RE + WA, and MAOC increased by 153% after WA and by 173% after RE + WA (Figure 3). As a result, the percentage of the POC fraction in the total SOC pool for low initial biocrust cover increased after 9 years of WA (increase in 30%) and RE + WA (13%), whereas that of MAOC decreased (18% with WA and 13% with RE + WA) (Table 1).

**Table 1:** Effects of Biocrusts and Climate Change on the Distribution of SOC Fractions

Initial biocrust cover	Climate treatment	POC at 0 years (%)	POC at 9 years (%)	MAOC at 0 years (%)	MAOC at 9 years (%)
Low	Control	40.6 ± 1.6	36.6 ± 1.9	53.9 ± 2.5	59.7 ± 1.8
	RE	40.0 ± 2.1	42.6 ± 1.6	57.3 ± 3.7	54.4 ± 2.4
	WA	40.9 ± 2.7	53.1 ± 3.8	56.1 ± 2.2	46.1 ± 3.6
	RE + WA	46.1 ± 2.7	52.1 ± 2.5	48.8 ± 1.5	42.3 ± 3.6
High	Control	44.8 ± 3.0	46.1 ± 1.9	50.5 ± 0.6	50.6 ± 2.7
	RE	46.5 ± 3.0	42.0 ± 2.9	51.4 ± 2.3	57.2 ± 0.9
	WA	44.4 ± 4.3	46.0 ± 4.0	56.8 ± 3.7	53.5 ± 3.1
	RE + WA	42.8 ± 2.8	55.7 ± 4.7	56.3 ± 2.6	44.1 ± 3.0

Percentage of organic C as particulate organic C (POC) and mineral-associated organic C (MAOC) (mean ± standard error, *n* = 5) in soils with low and high initial biocrust cover, after 0 and 9 years of ambient conditions (control), rainfall exclusion (RE), warming (WA), and the combination of both (RE + WA).

### Molecular Composition of Particulate Organic C

The molecular composition of the POC fraction in soils with low initial biocrust cover presented a less marked contribution of *O*-alkyl C compounds, an increased presence of *N*-alkyl and aromatic C, and greater alkyl/*O*-alkyl C ratios than that of the POC fraction in soils with high initial biocrust cover, for all the treatments examined. Regardless of initial biocrust cover, RE + WA, WA, and, to a lesser extent, RE decreased the aromaticity of POC and increased the *O*-alkyl contribution to its molecular composition with respect to the control treatment. In soils with low initial biocrust cover, the alkyl/*O*-alkyl C ratio of POC in soils under all climate manipulations was smaller than that of POC in the control soils (Table 2).

**Table 2:** Effects of Biocrusts and Climate Change on the Molecular Composition of POC

Initial biocrust cover	Climate treatment	Carboxylic C	Heteroaromatic C	Aromatic C	*O*-Alkyl C	*N*-Alkyl C	Alkyl C	Alkyl/*O*-alkyl C	Aromaticity
Low	Control	8.8	5.7	16.8	35.1	10.6	23.0	0.66	22.5
	RE	8.5	6.0	14.9	38.8	10.5	21.3	0.55	20.8
	WA	8.9	5.6	12.8	40.2	9.2	23.3	0.58	18.4
	RE + WA	8.9	5.2	12.4	40.6	9.6	23.3	0.57	17.6
High	Control	8.4	6.4	15.6	39.6	9.5	20.6	0.52	21.9
	RE	9.6	5.3	12.3	41.7	8.7	22.4	0.53	17.6
	WA	9.1	5.2	12.1	42.6	8.8	22.4	0.54	17.2
	RE + WA	8.3	4.5	10.6	43.0	9.1	24.5	0.57	15.1

^13^C NMR estimates of C structures of POC in soils under low and high initial biocrust cover after 9 years of ambient conditions (control), rainfall exclusion (RE), warming (WA), and the combination of both (RE + WA).

## Discussion

Understanding how biocrust communities regulate the response of SOC to climate change is essential to improve our ability to forecast C feedbacks in dryland ecosystems. In this study, we monitored SOC over 9 years of simulated warming and drought conditions to evaluate the influence of biocrust on the capacity of soils to store C. We found that the presence of biocrust cover modulates the effect of simulated climate change, resulting in an increase or no loss of SOC contents for low and high initial biocrust cover soils, respectively. However, this effect may be transitory since it is associated with the unprotected C and linked to an increase in carbohydrates and a decrease in aromatic compounds.

Carbon in the form of non-living biomass from biocrusts is incorporated into the soil and decomposed at a similar rate than plant litter in the study area (Berdugo and others 2021). An increase in the mortality of biocrust-forming lichens has been hypothesized to drive the increase in SOC observed after 4 years of warming in high initial biocrust cover areas in our experiment (Maestre and others 2013). However, this increase in SOC may disappear in the long-term as a consequence of progressive reduction in biocrust cover and lichen-derived soil C inputs. In fact, our results show that the positive warming effects on SOC observed in Maestre and others (2013) under high biocrust levels disappear after 9 years.

In contrast with the results for high initial biocrust cover areas, we found an increase in SOC associated with WA and RE + WA in soils with low initial biocrust cover after 9 years. This increase indicates that C inputs through the incorporation of non-living biocrust biomass into the soil (Berdugo and others 2021) are higher than soil C losses via soil respiration. Also, the cyanobacteria present in the area, which include common biocrust species from the genera *Microcoleus* and *Trichocoleus* (Cano-Díaz and others 2018), may have increased their abundance in detriment of that of lichens (as observed in similar experiments in other parts of the world; Ferrenberg and others 2015) providing additional C inputs into the soil. This result is also consistent with other studies on the same system indicating that soil respiration in areas with low initial biocrust cover is smaller than that in areas with high initial biocrust cover (Dacal and others 2020; Escolar and others 2015). In addition, our study did not detect a statistical effect of the single treatment of reduced rainfall (RE) on SOC, but significant effects of WA and RE + WA on this variable were observed. This is in line with previous studies in the same area showing that WA and RE + WA (but not RE alone) reduce soil respiration (Escolar and others 2015; Dacal and others 2020) and therefore C loss, probably because of a soil moisture reduction induced by an increased evaporation (Lafuente and others 2018).

To sum up, our observations of no net organic C gains in soils with high biocrust cover may be attributed to rapid losses of C inputs into the soil through high respiration rates. In contrast, low respiration of soils with low initial biocrust cover under WA treatments facilitates the preservation of biocrust-derived C inputs, increasing SOC (García-Palacios and others 2018; Dacal and others 2020). Future studies including soils with no biocrust cover would be useful to further disentangle biocrust cover effects on SOC. It must be noted that the potential contribution of plants to the effects on SOC described in our study may be arguably expected to be negligible. This is so because our experimental plots were established in the interspaces with sparse perennial cover (some small individuals and seedlings of the shrub *Helianthemum squamatum* are present across the plots) and the roots of the dominant plant species in the area (*Stipa tenacissima*) do not spread beyond their canopies (Puigdefábregas and others 1999). In fact, we did not visually detect plant roots in our soil samples.

The responses of biocrust communities to disturbances may be remarkably similar across large latitudinal and longitudinal gradients, even in cases where species identity and composition vary (Belnap and Lange 2003). Thus, the shifts in biocrust cover in response to climate change observed here may be reasonably expected to be found in other ecosystems under similar scenarios (Ferrenberg and other 2015; Finger-Higgens and others 2022). However, previous studies have also documented that biocrust composition, respiration and cover development highly depend on soil moisture (Chamizo and others 2016). This suggests that more research is needed to elucidate the potential effects of climate change in SOC and its fractions, especially in other environments with different soil moisture regimes.

The previous literature highlighted that in a scenario without biocrust, higher temperatures can enhance SOC decomposition (Conant and others 2011) and decrease C stocks (Link and others 2003). Across terrestrial ecosystems, most studies that have simulated warming found soil C losses associated with them (Crowther and others 2016). In our study, however, SOC remained constant or even increased after climate manipulations for high or low initial biocrust cover levels, respectively. This result indicates that the presence of biocrust communities reduces the negative effects of simulated climate change on SOC. Nevertheless, biocrust cover reductions associated with warming in our experiment (Dacal and others 2020; Ladrón de Guevara and others 2018) may compromise this effect in the long term, so the observed maintenance or increase of SOC associated with biocrust could be transitory.

The literature evaluating the effects of experimental warming and rainfall exclusion on soil C in biocrust-dominated drylands is very scarce and shows contrasting results, from negative (Darrouzet-Nardi and others 2015) to positive (García-Palacios and others 2018). We still need to improve our knowledge on the effects of warming and rainfall reduction on POC and MAOC fractions to better understand how and to what extent SOC is protected from decomposition in the context of climate change (Cotrufo and others 2019). The increment in SOC content with warming in low biocrust cover plots was observed for both the unprotected POC and mineral-protected MAOC fractions, but was particularly evident for the former. This is consistent with a higher responsiveness of the POC fraction to disturbances compared to MAOC. In contrast, previous studies have shown a decrease in SOC content with warming, especially in the POC fraction (He and others 2012; Link and others 2003; Song and others 2012). This decline has been attributed to an increase in microbial decomposition and respiration associated with elevated temperatures in mesic environments (Lehmann and others 2017; Rillig and others 2002). The higher turnover rate and sensitivity of the POC fraction to environmental changes compared to MAOC (He and others 2012; Link and others 2003; Song and others 2012) may explain the relatively higher increase in POC detected under the WA and RE + WA treatments and can be attributed to the incorporation of non-living lichen biomass from biocrusts (Berdugo and others 2021). Although less remarkable, the increase in MAOC observed may be attributed to warming causing a reduced turnover of non-living biomass due to suppression of fungal growth and soil respiration (Bai and others 2020).

The cover of biocrusts largely determines the amount of C transferred to the soil (Bowker and others 2018), and subsequently the SOC contents and its molecular composition through contributions as soil carbohydrates, polyphenols, and other complex aliphatic macromolecules (Miralles and others 2013; Xu and others 2021). Our compositional NMR results, with smaller alkyl/*O*-alkyl C ratios being indicative of lower decomposition of SOC (Baldock and others 1992), show that (1) Biocrusts could be sources of *O*-alkyl C and relatively fresh (undecomposed) input attributable to non-living biocrust to the POC fraction of soil, and that (2) Climate change, especially warming and the combination of warming and rainfall exclusion, may lead to a relative increase in carbohydrates compensated by a relative decrease in aromatic compounds in soils with low biocrust cover. Maestre and others (2013) registered in our experiment an increase in phenols with warming 46 months after the beginning of the experiment and suggested that this response could be driven by increased lichen mortality under warming, which increased the sources of recalcitrant compounds via their decomposition. Even though our results are not aligned with these, polyphenolic compounds, such as lignin, usually bind to mineral surfaces (Thevenot and others 2010), so the increase in recalcitrant compounds may be reflected in the MAOC fraction. Therefore, NMR data show a trade-off between aromatic C and *O*-alkyl C contribution to the molecular structures of POC for all treatments. Biocrusts can mitigate the effect of climate change on organic matter degradation status by providing larger inputs of fresh organic materials to soils. However, over a longer period, rainfall exclusion and especially warming may cause the death of lichens from biocrusts, adding inputs of fresh and labile *O*-alkyl C, mostly carbohydrates, that are expected to be degraded. The compositional changes observed in the POC fraction in low initial biocrust cover areas point to a lower decomposition degree that, together with the observed POC accumulation, suggests that the increases in SOC observed in the WA and WA + RE treatments will likely not persist in the long term.

## Conclusions

Overall, our results indicate that biocrusts may prevent SOC losses associated with climate change in drylands. These findings, based on a 9-year experiment and the assessment of physical and biochemical protection mechanisms of SOC, indicate that the modulation effect of biocrusts may be mainly attributed to the increased C inputs of non-living biocrust to the relatively more highly degradable and vulnerable fraction of SOC. Our data suggest that the observed increases of SOC under warming can be a transitory response because they are driven by the accumulation of labile C fractions, while biocrust cover is detrimentally affected at the same time by warming. Future research needs to unveil the persistence of this effect. This will be critical to better understand and manage the impacts and consequences of climate change on the multiple functions and ecosystem services provided by SOC in biocrust-dominated drylands.

## Supplementary Information

Below is the link to the electronic supplementary material.

Supplementary file1 (RTF 13808 kb).

Supplementary file2 (DOCX 12 kb).

## Data Availability

The data associated with this study are publicly available in Zenodo (https://zenodo.org/record/1313544#.YaYOzdDMJaQ).

## References

[CR1] Ahlström A, Raupach MR, Schurgers G, Smith B, Arneth A, Jung M, Reichstein M, Canadell JG, Friedlingstein P, Jain AK, Kato E, Poulter B, Sitch S, Stocker BD, Viovy N, Wang YP, Wiltshire A, Zaehle S, Zeng N (2015). The dominant role of semi-arid ecosystems in the trend and variability of the land CO_2_ sink. Science.

[CR2] Bai T, Wang P, Hall SJ, Wang F, Ye C, Li Z, Li S, Zhou L, Qiu Y, Guo J, Guo H, Wang Y, Hu S (2020). Interactive global change factors mitigate soil aggregation and carbon change in a semi-arid grassland. Global Change Biology.

[CR3] Baldock JA, Smernik RJ (2002). Chemical composition and bioavailability of thermally altered Pinus resinosa (Red pine) wood. Organic Geochemestry.

[CR4] Baldock JA, Oades JM, Nelson PN, Skene TM, Golchin A, Clarke P (1992). Assessing the extent of decomposition of natural organic materials using solid-state ^13^C NMR spectroscopy. Australian Journal of Soil Research.

[CR5] Bates D, Maechler M, Bolker B, Walker S. 2015. lme4: Linear mixed-effects models using Eigen and S4 package version 1.1–7. 2014.

[CR6] Belnap J, Lange O. 2003. Biological soil crusts: structure, function, and management, vol 150, Ecological studies. Springer, Heidelberg 2nd edition.

[CR7] Belnap J, Weber B, Büdel B. 2016. Biological Soil Crusts as an Organizing Principle in Drylands. Springer 3–13.

[CR8] Berdugo M, Delgado-Baquerizo M, Soliveres S, Hernández-Clemente R, Zhao Y, Gaitán JJ, Gross N, Saiz H, Maire V, Lehman A, Rillig MC, Solé RV, Maestre FT (2020). Global ecosystem thresholds driven by aridity. Science.

[CR9] Berdugo M, Mendoza-Aguilar DO, Rey A, Ochoa V, Gozalo B, García-Huss L, Maestre FT. 2021. Litter decomposition rates of biocrust-forming lichens are similar to those of vascular plants and are affected by warming. Ecosystems 1–14.

[CR10] Bowker MA, Reed SC, Maestre FT, Eldridge DJ (2018). Biocrusts: the living skin of the earth. Plant and Soil.

[CR11] Bradford MA, Keiser AD, Davies CA, Mersmann CA, Strickland MS (2013). Empirical evidence that soil carbon formation from plant inputs is positively related to microbial growth. Biogeochemistry.

[CR12] Cano-Díaz C, Mateo P, Muñoz-Martín MÁ, Maestre FT (2018). Diversity of biocrust-forming cyanobacteria in a semiarid gypsiferous site from Central Spain. Journal of Arid Environments.

[CR13] Castillo-Monroy AP, Maestre FT, Rey A, Soliveres S, García-Palacios P (2011). Biological Soil Crust Microsites Are the Main Contributor to Soil Respiration in a Semiarid Ecosystem. Ecosystems.

[CR15] Chamizo S, Rodríguez-Caballero E, Moro MJ, Cantón Y (2021). Non-rainfall water inputs: A key water source for biocrust carbon fixation. Science of the Total Environment.

[CR16] Chamizo S, Belnap J, Eldridge D, Cantón Y, Issa O M. 2016. The role of biocrusts in arid land hydrology. Weber B, Büdel B, Belnap J, editors. Biological soil crusts: an organizing principle in drylands. Springer International Publishing. p21–346.

[CR17] Cherlet M, Hutchinson C, Reynolds J, Hill J, Sommer S, Maltitz G (2018). World atlas of desertification In Luxembourg.

[CR18] Conant RT, Ryan MG, Ågren GI, Birge HE, Davidson EA, Eliasson PE, Evans SE, Frey SD, Giardina CP, Hopkins FM, Hyvönen R, Kirschbaum MUF, Lavallee JM, Leifeld J, Parton WJ, Megan Steinweg J, Wallenstein MD, Wetterstedt JÅ, Bradford MA (2011). Temperature and soil organic matter decomposition rates – synthesis of current knowledge and a way forward. Global Change Biology.

[CR19] Concostrina-Zubiri L, Valencia E, Ochoa V, Gozalo B, Mendoza BJ, Maestre FT (2021). Species-specific effects of biocrust-forming lichens on soil properties under simulated climate change are driven by functional traits. In New Phytologist.

[CR20] Cotrufo MF, Lavallee JM (2022). Soil organic matter formation, persistence, and functioning: A synthesis of current understanding to inform its conservation and regeneration. Advances in Agronomy.

[CR22] Cotrufo MF, Ranalli MG, Haddix M L, Six J, Lugato E. 2019. Soil carbon storage informed by particulate and mineral-associated organic matter. Nature Geoscience 1–6.

[CR23] Crowther TW, O Todd-brown KE, Rowe W, Wieder WR, Carey J, Snoek L, Fang S, Zhou G, Allison S, Blair J, Bridgham S, Burton AJ, Carrillo Y, Reich P, Clark JS, Classen AT, Dijkstra FA, Emmett A, Frey S, Guo J, Harte J, Jiang L, Johnson BR, Kröel-Dulay G, Larsen KS, Laudon H, Lavallee JM, Luo Y, Lupascu M, Ma LN, Marhan S, Michelsen A, Mohan J, Niu S, Pendall E, Peñuelas J, Pfeifer-Meister L, Poll C, Reinsch S, Reynolds LL, Schmidt IK, Sistla S, Sokol NW, Templer PH, Treseder KK, Welker JM, Bradford MA. 2016. Quantifying global soil carbon losses in response to warming. Nature 540 10.1038/nature20150 27905442

[CR24] Dacal M, García-Palacios P, Asensio S, Cano-Díaz C, Gozalo B, Ochoa V, Maestre FT (2020). Contrasting mechanisms underlie short-and longer-term soil respiration responses to experimental warming in a dryland ecosystem. Global Change Biology.

[CR25] Darrouzet-Nardi A, Reed SC, Grote EE, Belnap J (2015). Observations of net soil exchange of CO2 in a dryland show experimental warming increases carbon losses in biocrust soils. Biogeochemistry.

[CR26] Davidson EA, Janssens IA (2006). Temperature sensitivity of soil carbon decomposition and feedbacks to climate change. Nature.

[CR14] De Castro M, Martín-Vide J, Alonso S. 2005. El clima de España: pasado, presente y escenarios de clima para el siglo XXI.

[CR27] Delgado-Baquerizo M, Maestre FT, Reich PB, Trivedi P, Osanai Y, Liu YR, Hamonts K, Jeffries TC, Singh BK (2016). Carbon content and climate variability drive global soil bacterial diversity patterns. Ecological Monographs.

[CR28] Dinno A, Dinno AM (2017). Package ‘dunn. test’. CRAN Repos.

[CR29] Elbert W, Weber B, Burrows S, Steinkamp J, Büdel B, Andreae MO, Pöschl U (2012). Contribution of cryptogamic covers to the global cycles of carbon and nitrogen. Nature Geoscience.

[CR30] Escolar C, Martínez I, Bowker MA, Maestre FT (2012). Warming reduces the growth and diversity of biological soil crusts in a semi-arid environment: Implications for ecosystem structure and functioning. Philosophical Transactions of the Royal Society b: Biological Sciences.

[CR31] Escolar C, Maestre FT, Rey A (2015). Biocrusts modulate warming and rainfall exclusion effects on soil respiration in a semi-arid grassland. Soil Biology and Biochemistry.

[CR32] FAO I. 2015. Status of the world’s soil resources (SWSR)–main report. Food and agriculture organization of the United Nations and intergovernmental technical panel on soils, Rome, Italy, 650.

[CR33] Feng S, Fu Q (2013). Expansion of global drylands under a warming climate. Atmospheric Chemistry and Physics.

[CR34] Ferrenberg S, Reed SC, Belnap J, Schlesinger WH (2015). Climate change and physical disturbance cause similar community shifts in biological soil crusts. Proceedings of the National Academy of Sciences of the United States of America.

[CR35] Finger-Higgens R, Duniway MC, Fick S, Geiger EL, Hoover DL, Pfennigwerth AA, Van Scoyoc MW, Belnap J. 2022. Decline in biological soil crust N-fixing lichens linked to increasing summertime temperatures. Proceedings of the National Academy of Sciences 119(16). 10.1073/pnas.2120975119 PMC9169860 35412916

[CR36] García-Palacios P, Escolar C, Dacal M, Delgado-Baquerizo M, Gozalo B, Ochoa V, Maestre FT (2018). Pathways regulating decreased soil respiration with warming in a biocrust-dominating dryland. Global Change Biology.

[CR37] García-Palacios P, Gross N, Gaitán JJ, Maestre FT (2018). Climate mediates the biodiversity–ecosystem stability relationship globally. Proceedings of the National Academy of Sciences of the United States of America.

[CR38] Golchin A, Oades JM, Skjemstad JO, Clarke P (1994). Study of free and occluded particulate organic matter in soils by solid state ^13^C CP/MAS NMR spectroscopy and scanning electron microscopy. Soil Research.

[CR39] Groffman PM, Driscoll CT, Fahey TJ, Hardy JP, Fitzhugh RD, Tierney GL (2001). Colder soils in a warmer world: A snow manipulation study in a northern hardwood forest ecosystem. Biogeochemistry.

[CR40] Harris D, Horwáth WR, van Kessel C (2001). Acid fumigation of soils to remove carbonates prior to total organic carbon or CARBON-13 isotopic analysis. Soil Science Society of America Journal.

[CR41] He N, Chen Q, Han X, Yu G, Li L (2012). Warming and increased procipitation individually influence soil carbon sequestration of Inner Mongolian grasslands, China. Agriculture, Ecosystems & Environment.

[CR42] Huang J, Yu H, Guan X, Wang G (2016). Accelerated dryland expansion under climate change. Nature Climate Change.

[CR43] IUSS Working Group WRB. 2015. World Reference Base for Soil Resources 2014, update 2015. World Soil Resources Reports No. 106. FAO, Rome, Italy.

[CR44] Kleber M, Eusterhues K, Keiluweit M, Mikutta C, Mikutta R, Nico PS (2015). Mineral-Organic Associations: Formation, Properties, and Relevance in Soil Environments. Advances in Agronomy.

[CR45] Kleber M, Bourg IC, Coward EK, Hansel CM (2021). Dynamic interactions at the mineral–organic matter interface. Nature Reviews Earth & Environment.

[CR46] Knicker H (2011). Solid state CPMAS ^13^C and ^15^N NMR spectroscopy in organic geochemistry and how spin dynamics can either aggravate or improve spectra interpretation. Organic Geochemistry.

[CR47] Kuznetsova A, Brockhoff PB, Christensen RHB (2015). Package ‘lmertest’. R Package Version.

[CR48] Ladrón de Guevara M, Gozalo B, Raggio J, Lafuente A, Prieto M, Maestre FT (2018). Warming reduces the cover, richness and evenness of lichen-dominated biocrusts but promotes moss growth: insights from an 8 yr experiment. New Phytologist.

[CR49] Lafuente A, Berdugo M, Gozalo B, Maestre FT (2018). Simulated climate change affects how biocrusts modulate water gains and desiccation dynamics after rainfall events. Ecohydrology.

[CR50] Lavallee JM, Soong JL, Cotrufo MF (2020). Conceptualizing soil organic matter into particulate and mineral - associated forms to address global change in the 21st century. Global Change Biology.

[CR51] Lehmann A, Zheng W, Rillig MC (2017). Soil biota contributions to soil aggregation. Nature Ecology & Evolution.

[CR52] Liang C, Amelung W, Lehmann J, Kästner M (2019). Quantitative assessment of microbial necromass contribution to soil organic matter. Global Change Biology.

[CR53] Link SO, Smith JL, Halvorson JJ, JrH Bolton (2003). A reciprocal transplant experiment within a climatic gradient in a semiarid shrub-steppe ecosystem: effects on bunchgrass growth and reproduction, soil carbon, and soil nitrogen. Global Change Biology.

[CR54] Lüdemann HD, Nimz H (1973). Carbon-13 nuclear magnetic resonance spectra of lignins. Biochemical and Biophysical Research Communications.

[CR55] Maestre FT, Escolar C, Ladrón de Guevara M, Quero JL, Lázaro R, Delgado-Baquerizo M, Ochoa V, Berdugo M, Gozalo B, Gallardo A (2013). Changes in biocrust cover drive carbon cycle responses to climate change in drylands. Global Change Biology.

[CR56] Maestre FT, Delgado-Baquerizo M, Jeffries TC, Eldridge DJ, Ochoa V, Gozalo B, Quero JL, García-Gómez M, Gallardo A, Ulrich W, Bowker MA, Arredondo T, Barraza-Zepeda C, Bran D, Florentino A, Gaitán JJ, Gutiérrez JR, Huber-Sannwald E, Jankju M, Mau RL, Miriti M, Naseri K, Ospina A, Stavi I, Wang D, Woods AA, Yuan X, Zaady E, Singh BK (2015). Increasing aridity reduces soil microbial diversity and abundance in global drylands. Proceedings of the National Academy of Sciences.

[CR57] Maestre FT, Benito BM, Berdugo M, Concostrina‐Zubiri L, Delgado‐Baquerizo M, Eldridge DJ, Guirado E, Gross N, Kéfi S, Le Bagousse‐Pinguet Y, Ochoa‐Hueso R, Soliveres S. 2021. Biogeography of global drylands. New Phytologist. 10.1111/nph.17395 33864276

[CR58] Mager DM, Thomas AD (2011). Extracellular polysaccharides from cyanobacterial soil crusts: A review of their role in dryland soil processes. Journal of Arid Environments.

[CR59] Miralles I, Trasar-Cepeda C, Leirós MC, Gil-Sotres F (2013). Labile carbon in biological soil crust in the Tabernas desert, SE Spain. Soil Biology and Biochemistry.

[CR61] Pedersen TL. 2020. Patchwork: The Composer of Plots.

[CR62] Plaza C, Gascó G, Méndez AM, Zaccone C, Maestre FT. 2018. Chapter 2 – Soil Organic Matter in Dryland Ecosystems. Garcia C, Nannipieri P, Hernandez T. editors. In The Future of Soil Carbon. Elsevier Inc. p39–70.

[CR63] Poeplau C, Don A, Six J, Kaiser M, Benbi D, Chenu C, Cotrufo MF, Derrien D, Gioacchini P, Grand S, Gregorich E, Griepentrog M, Guninano A, Haddix M, Kuzyakov Y, Kühnel A, Macdonald LM, Soong J, Trigalet S, Vermeire M-L, Rovira P, Wesemael M, Wiesmeier M, Yeasmin S, Yevdokimov I, Nieder R (2018). Isolating organic carbon fractions with varying turnover rates in temperate agricultural soils–A comprehensive method comparison. Soil Biology and Biochemistry.

[CR65] Puissant J, Mills RTE, Robroek BJM, Gavazov K, Perrette Y, De Danieli S, Spiegelberger T, Buttler A, Brun JJ, Cécillon L (2017). Climate change effects on the stability and chemistry of soil organic carbon pools in a subalpine grassland. Biogeochemistry.

[CR66] R Core Team .2021. R: A language and environment for statistical computing. R Foundation for Statistical Computing, Vienna, Austria.

[CR67] Reed SC, Delgado-Baquerizo M, Ferrenberg S (2019). Biocrust science and global change. New Phytologist.

[CR68] Rillig MC, Wright SF, Shaw MR, Field CB (2002). Artificial climate warming positively affects arbuscular mycorrhizae but decreases soil aggregate water stability in an annual grassland. Oikos.

[CR69] Rodriguez-Caballero E, Belnap J, Büdel B, Crutzen PJ, Andreae MO, Pöschl U, Weber B (2018). Dryland photoautotrophic soil surface communities endangered by global change. Nature Geoscience.

[CR70] Safriel U, Adeel Z, Niemeijer D, Puigdefabregas J, White R, Lal R, Winslow M, Ziedler J, Prince S, Archer E, King C, Shapiro B, Wessels K, Nielsen TT, Portnov B, Reshef I, Thornell J, Lachman E, McNab D. 2005. Dryland systems. Hassan R, Scholes R, Ash N, editors. In Ecosystems and Human Well-being: Current State and Trends.: Findings of the Condition and Trends Working Group. Island Press. pp. 623–662.

[CR71] Schnecker J, Borken W, Schindlbacher A, Wanek W (2016). Little effects on soil organic matter chemistry of density fractions after seven years of forest soil warming. Soil Biology and Biochemistry.

[CR72] Song B, Niu S, Zhang Z, Yang H, Li L, Wan S (2012). Light and heavy fractions of soil organic matter in response to climate warming and increased precipitation in a temperate steppe. PloS One.

[CR73] Thevenot M, Dignac MF, Rumpel C (2010). Fate of lignins in soils: a review. Soil Biology and Biochemistry.

[CR74] Thomas AD (2012). Impact of grazing intensity on seasonal variations in soil organic carbon and soil CO_2_ efflux in two semiarid grasslands in southern Botswana. Philosophical Transactions of the Royal Society b: Biological Sciences.

[CR75] Wander F. 2004. Chapter 3: Soil Organic Matter Fractions and Their Relevance to Soil Function. Magdoff B, Weil RR, editors. Soil Organic Matter in Sustainable Agriculture. 1st Edition. ImprintCRC Press. p36.

[CR76] Wang Y, Li X, Wu X, Hong Y, Wang T, Zuo F, Zhang J, Yang X (2021). Divergent effects of biological soil crusts on soil respiration between bare patches and shrub patches under simulated rainfall in a desert ecosystem in Northwest China. Soil and Tillage Research.

[CR77] Wickham H (2017). The Tidyverse. R Package Ver..

[CR78] Wickham H, Bryan J, Kalicinski M, Valery K, Leitienne C, Colbert B, Hoerl D, Miller E, Bryan MJ. 2019. Package “readxl.”

[CR79] Wood SA, Sokol N, Bell CW, Bradford MA, Naeem S, Wallenstein MD, Palm CA (2016). Opposing effects of different soil organic matter fractions on crop yields. Ecological Applications.

[CR80] Xu H, Zhang Y, Shao X, Liu N (2021). Soil nitrogen and climate drive the positive effect of biological soil crusts on soil organic carbon sequestration in drylands: A Meta-analysis. Science of the Total Environment.

[CR81] Yahdjian L, Sala OE (2002). A rainout shelter design for intercepting different amounts of rainfall. Oecologia.

