# Supplementary material for: Biocrusts Modulate Climate Change Effects on Soil Organic Carbon Pools: Insights From a 9-Year Experiment
Source: Ecosystems. 2022 Sep 27;26(3):585–96. doi: 10.1007/s10021-022-00779-0 (PMC10167156; doi:10.1007/s10021-022-00779-0)
Supplement: Supplementary file 1 — Supplementary file1 (RTF 13808 kb). [file 10021_2022_779_MOESM1_ESM.rtf]

Supplementary material for Díaz-Martínez et al. “Biocrusts buffer against climate change effects on soil organic carbon pools: Insights from a 9-year experiment”

Authors: Paloma Díaz-Martínez, Marco Panettieri, Pablo García-Palacios, Eduardo Moreno, César Plaza, Fernando T. Maestre


Table of contents:
Supplementary table 1. Linear mixed effect model for soil organic C after 9 years.

Extended data fig. 1. Detailed view of an experimental plot with an open top chamber (OTC, b), a plot with a rainfall shelter (d) and, two plots with an OTC and a rainfall shelter (a and c). Methacrylate grooves are connected to plastic bottles that accumulate the excluded water. 


Supplementary table 1. Linear mixed effect model for soil organic C after 9 years.
	Estimate	std error	t-value	p-value	
Intercept	10.70	3.63	2.95	0.0060	
RE	3.90	5.14	0.76	0.4536	
WA	14.19	5.14	2.76	0.0094	
RE+WA	17.00	5.14	3.31	0.0023	
HIBC	12.45	5.14	2.42	0.0212	
RE:HIBC	-9.83	7.26	-1.35	0.1854	
WA:HIBC	-16.31	7.26	-2.25	0.0318	
RE+WA:HIBC	-14.09	7.26	-1.94	0.0614	
Standard error (std error), rainfall exclusion (RE), warming (WA), and the combination of both (RE+WA), high initial biocrust cover (HIBC). Significant values are highlighted in bold. 


Extended data fig. 1. Detailed view of an experimental plot with an open top chamber (OTC, b), a plot with a rainfall shelter (d) and, two plots with an OTC and a rainfall shelter (a and c). Methacrylate grooves are connected to plastic bottles that accumulate the excluded water. 
