# Supplementary material for: Biocrusts Modulate Climate Change Effects on Soil Organic Carbon Pools: Insights From a 9-Year Experiment
Source: Ecosystems. 2022 Sep 27;26(3):585–96. doi: 10.1007/s10021-022-00779-0 (PMC10167156; doi:10.1007/s10021-022-00779-0)
Supplement: Supplementary file 2 — Supplementary file2 (DOCX 12 kb). [file 10021_2022_779_MOESM2_ESM.docx]

ECOSYSTEMS MANUSCRIPT INFORMATION SHEET

MANUSCRIPT NUMBER: ECO-22-0031.R3

TITLE: Biocrusts modulate climate change effects on soil organic carbon pools: Insights from a 9-year experiment

AUTHORS: Díaz Martínez, Paloma; Panettieri, Marco; Garcia-Palacios, P.; Moreno, Eduardo; Plaza, Cesar; Maestre, Fernando

CORRESPONDING AUTHOR:

Dr. Paloma Díaz Martínez

Departamento de Biología y Geología, Física y Química Inorgánica Universidad Rey Juan Carlos Madrid 28933 Spain

FAX:

PHONE:

EMAIL: [paloma.diaz@urjc.es](mailto:paloma.diaz@urjc.es)

RECEIVED 28-Jan-2022; ACCEPTED 14-Jul-2022

COLOR FIGURES: 3

COMMENTS:
